# Development and validation of a predictive nomogram for early detection of necrotizing pneumonia in children with refractory *Mycoplasma pneumoniae* pneumonia

**DOI:** 10.3389/fped.2026.1725447

**Published:** 2026-01-29

**Authors:** Jianqin Zhang, Li Cheng, Shujun Jing, Jiaohui Fu, Haixia Chen, Kejia Xiao, Yuxia Shan

**Affiliations:** Dalian Women and Children’s Medical Center (Group), Dalian, China

**Keywords:** children, infection, mycoplasma pneumonia, necrotizing pneumonia, nomogram, pneumonia

## Abstract

**Objective:**

To identify predictive factors for necrotizing pneumonia (NP) in children with refractory *Mycoplasma pneumoniae pneumonia* (RMPP) and develop a predictive nomogram.

**Methods:**

A retrospective analysis was conducted on clinical data of children with RMPP admitted to the Affiliated Women and Children's Hospital of Dalian University of Technology between June 2023 and July 2024. The dataset was randomly split into a training set (70%, *n* = 197) for model development and a test set (30%, *n* = 77) for internal validation. The *χ*^2^ test and Mann–Whitney *U* test were used to screen potential predictors, and multivariate logistic regression analysis was applied to establish a clinical prediction model. The Hosmer-Lemeshow test was used to evaluate model fit, and variance inflation factor was calculated to assess multicollinearity. The discriminatory and calibrative performance of the nomogram was evaluated using the receiver operating characteristic (ROC) curve and calibration curve, respectively.

**Results:**

A total of 274 children with RMPP were analyzed. Of these, 51 who developed NP formed the necrotizing group, while the remaining 223 without NP were designated as the non-necrotizing group. The *χ*^2^ text and Mann–Whitney *U* Test analysis indicated that ESR, CD4^+^ *T* cells, NK cells, IL−4, duration of fever, and pleural effusion were significant predictors of NP in children with MPP (*P* < 0.05). Internal validation using the test set showed a consistency rate of 87.01% (67/77) between predicted and actual outcomes. The model demonstrated a sensitivity of 0.833, specificity of 0.877, and a Kappa coefficient of 0.590. Although predictive accuracy slightly decreased in the test set compared to the training set, the model still retained satisfactory predictive performance, indicating its potential generalizability.

**Conclusion:**

The prediction model incorporating ESR, CD4^+^ T cells, NK cells, IL-4, duration of fever, and pleural effusion showed good predictive value for NP in children with RMPP.

## 1 Introduction

*Mycoplasma pneumoniae* (MP), a major pathogen responsible for community-acquired pneumonia in children, accounts for approximately 10%–40% of community-acquired pneumonia cases (1). *Mycoplasma pneumoniae* pneumonia (MPP), previously regarded as a self-limiting illness with favorable outcomes, typically presents in children with fever and persistent dry cough (2, 3). However, some cases could develop into refractory *Mycoplasma pneumoniae* pneumonia (RMPP) complicated by serious intrapulmonary and extrapulmonary complications, including necrotizing pneumonia (NP), pulmonary embolism, central nervous system inflammation, acute myocardial injury, and thrombosis (4, 5).

NP, alternatively termed cavitary pneumonia or cavitary necrosis, is characterized by massive necrosis and liquification of lung tissues (6, 7). Although lacking a universally standardized definition, NP is definitively diagnosed via chest CT as the gold standard, with key imaging features including destruction of lung parenchyma, liquefactive necrosis, and cavity formation (8). These findings are frequently accompanied by empyema and bronchopleural fistula. Although rare in pediatric MPP cases, NP may suddenly manifest as hydropneumothorax and more severe dyspnea, potentially life-threatening conditions (9). Dyspnea in NP is frequently more pronounced and is closely associated with the severe local complications characteristic of NP, such as substantial pleural effusion or hydropneumothorax, rather than being a non-specific finding. Diagnosing NP often involves a significant delay, with an average reported time of 17 days from symptom onset to imaging confirmation (10). This delay can postpone crucial interventions, underscoring the critical need for early predictive indicators. Consequently, identifying reliable predictors for NP in pediatric MPP has become a key focus of research.

This study aimed to identify predictive factors for NP development in children with RMPP with extensive pulmonary lesions. We retrospectively reviewed 274 children with RMPP admitted between June 2023 and July 2024, then systematically compared demographics, clinical presentations, laboratory parameters, and imaging features between NP and non-NP (NNP) groups. Characterization of NP's clinical trajectory and outcomes provides critical insights for early risk stratification and timely intervention to mitigate complications.

## 2 Methods

### 2.1 Patients

This single-center retrospective study analyzed data from pediatric patients diagnosed with MPP at the Affiliated Women and Children's Hospital of Dalian University of Technology (Dalian, China) between June 2023 and July 2024. The diagnosis of MPP was established based on the following criteria: clinical symptoms and signs suggestive of pneumonia, including fever, expectoration or wheezing, and abnormal lung auscultation; chest imaging demonstrating inflammatory infiltration; and either a single serum MP antibody titer exceeding 1:160 or a positive MP-DNA PCR test from nasopharyngeal aspirate, bronchoalveolar lavage fluid, or pleural effusion (11). The diagnostic criteria for RMPP included persistent fever and/or radiographic deterioration after seven days of initial macrolide antibiotic therapy (12). The inclusion and diagnostic criteria for NP comprised previously normal lung imaging, a confirmed diagnosis of MPP, and early chest CT findings (13).

Although complete anthropometric data for precise BMI calculation were not available for all participants, clinical records and physician assessments at admission indicated no cases of malnutrition among the included children. It was acknowledged that some participants were clinically documented as overweight or obese. All patients underwent routine preliminary serological screening, including tests for hepatitis B surface antigen (HBsAg), hepatitis C virus antibody (HCV-Ab), treponema pallidum antibody (TP-Ab), and human immunodeficiency virus (HIV) upon admission, and all results were negative.

This study complied with the Declaration of Helsinki and was approved by the Ethics Review Committee of the Women and Children's Hospital of Dalian University of Technology.

### 2.2 Data collection

Clinical data encompassed the following dimensions: baseline characteristics (sex, age), clinical signs (duration of fever and peak body temperature), laboratory parameters [white blood cell count (WBC), neutrophil percentage (NEUT%), C-reactive protein (CRP), erythrocyte sedimentation rate (ESR), D-dimer (DD), procalcitonin (PCT), ferritin, alanine aminotransferase (ALT), aspartate aminotransferase (AST), lactate dehydrogenase (LDH), prealbumin (PA), albumin (ALB), total protein (TP), IgE, CD4^+^ cells, CD8^+^ cells, CD19^+^ cells, NK cells, IL-4, IL-6, IL-10, Interferon gamma (γ-IFN)], and imaging manifestations (pulmonary consolidation, bronchoscopic plastic secretion, number of affected lung lobes).

These predictive parameters selected were grounded in a tripartite foundation comprising established clinical experience, supporting literature evidence (14–16), and recognized pediatric pneumonia guidelines (17), all of which consistently highlight the crucial roles of excessive inflammatory response and secondary tissue damage in the pathogenesis of NP. This coherent body of evidence provided a solid rationale for our focus on inflammatory, tissue-damage, and coagulation-related biomarkers.

For the analyzed variables in this study (including ESR, cellular immune parameters, etc.), the rate of missing data was below 5%. For sporadic missing values in specific indicators, a complete-case analysis approach was employed, meaning records with missing data for a particular variable were excluded from analyses involving that variable. All included cases contained complete data necessary for determining the primary outcome (development of necrotizing pneumonia).

### 2.3 Statistical analysis

Statistical analyses were conducted using IBM SPSS Statistics, Version 25.0 (IBM Corp., Armonk, NY, USA). Comprehensive comparisons between NP and NNP groups encompassed general characteristics, hematological parameters, hepatic/renal functions, immunological and cytokine profiles, and imaging manifestations. Categorical variables including gender, pulmonary consolidation, plastic bronchitis, and inflammatory stenosis were analyzed using *χ*^2^ tests. Non-normally distributed continuous variables such as age, hospitalization duration, and fever duration underwent Mann–Whitney *U* tests.

Prior to multivariate analysis, the dataset was randomly partitioned into training (70%, *n* = 197) and validation (30%, *n* = 77) sets. Multivariable logistic regression with forward likelihood ratio method was employed to develop an NP prediction model, incorporating statistically significant variables from univariate analyses. Model integrity was verified through Hosmer-Lemeshow goodness-of-fit testing and variance inflation factor (VIF) assessment for multicollinearity. Predictive performance was evaluated using receiver operating characteristic (ROC) curve analysis, with statistical significance defined as *P* < 0.05 throughout the study.

## 3 Results

### 3.1 Patient characteristics

**Figure 1:**
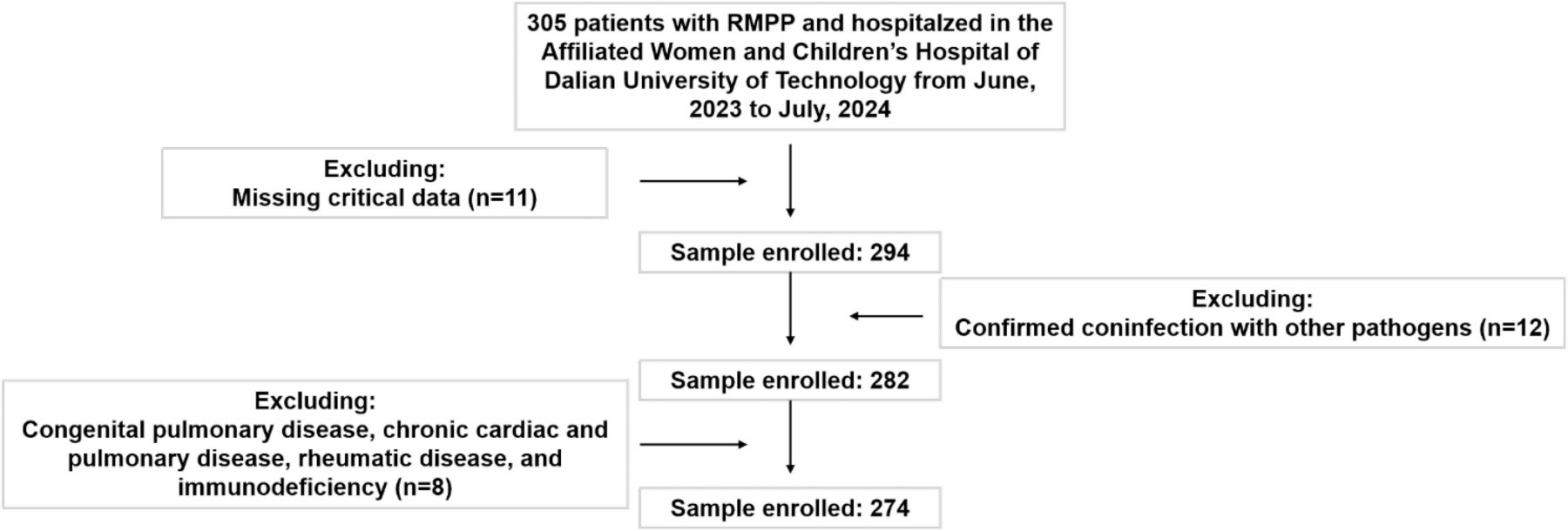
Patient screening flowchart. Inclusion and exclusion of children with refractory RMPP in the retrospective cohort (June 2023–July 2024).

From the initial cohort of 305 children meeting these RMPP diagnostic criteria, 31 cases were excluded through a systematic screening process: 11 due to missing critical data, 12 for confirmed co-infection with other pathogens, and 8 for congenital pulmonary disease, chronic cardiac and pulmonary disease, rheumatic diseases, and immunodeficiency. The final analytical cohort comprised 274 children, including 51 with NP and 223 with NNP (Figure 1). All patients tested positive by both PCR and serological assays and met exclusion criteria. Based on chest CT findings, patients were stratified into NP and NNP groups. The clinical characteristics of NP and NNP groups were summarized in Table 1. The NNP group comprised 223 patients (81.4%), with a median age of 8.0 (7.1–9.0) years. The NP group included 51 patients (18.6%), also with a median age of 8.0 (7.4–9.0) years. No statistically significant differences were observed between groups regarding gender or age distribution. However, the NP group exhibited significantly higher median values for length of hospital stay, duration of fever, and peak body temperature compared to the NNP group (all *P* *<* 0.001).

**Table 1:** Baseline characteristics of children with RMPP in the NP group and NNP group.

Clinical information	NP group (*n* = 51)	NNP group (*n* = 223)	*χ*^2^/*Z* value	*P* value
Sex^a^			3.324	0.068
Male	31 (42.11)	138 (50.36)		
Female	20 (57.89)	136 (49.64)		
Age (years)^b^	8.0 (7.4–9.0)	8.0 (7.1–9.0)	−0.493	0.622
Length of hospitalization (d)^b^	16.0 (12.0–24.5)	7.0 (6.0–8.5)	−8.522	<0.001
Length of fever (d)^b^	11.0 (8.0–13.0)	7.0 (6.0–9.0)	−6.435	<0.001
Maximum temperature (℃)^b^	39.8 (39.5–40.3)	39.4 (39.0–39.8)	−4.679	<0.001

Gender is presented as a fundamental demographic characteristic; Length of hospitalization is presented as an indirect indicator of disease severity and healthcare resource utilization.

^a^ Represented that categorical data were described as number of cases (*n*) and proportion (%), and group comparisons were performed using the *χ*^2^ test.

^b^ Represented that continuous data with non-normal distribution were described as the median (25th–75th percentile), and the Mann–Whitney *U* test was used for group comparisons.

### 3.2 Laboratory characteristics

Comparisons of laboratory parameters between the NP and NNP groups were presented in Table 2. Univariate analysis revealed that the NP group exhibited significantly higher median levels of neutrophil %, CRP, ESR, D-dimer, PCT, and ferritin (all *P* *<* 0.001), yet showed no significant difference in median WBC count compared to the NNP group. Regarding liver markers, the NP group demonstrated significantly elevated median levels of ALT, AST, and LDH (all *P* *<* 0.001), but significantly lower median levels of PAB, ALB, and TP (all *P* < 0.05). In immunological profiles, the NP group had significantly lower median levels of CD4^+^, CD8^+^, CD19^+^, and NK cells (all *P* *<* 0.05), yet significantly higher median levels of IL-4 (*P* *=* 0.023), IL-10, and IFN-γ (all *P* *<* 0.05). Importantly, no statistically significant differences were observed in median IgE or IL-6 between the groups.

**Table 2:** Laboratory findings of children with RMPP in the NP group and NNP group.

Laboratory data	NP group (*n* = 51)	NNP group (*n* = 223)	*Z* value	*P* value
Routine blood test parameters				
WBC (×10^9^ /L)	8.62 (6.45–12.97)	7.91 (6.60–10.17)	−1.705	0.088
NEUT% (%)	76.70 (68.00–85.80)	64.65 (57.38–72.83)	−5.680	<0.001
CRP (mg/L)	54.58 (38.00–90.75)	14.06 (6.12–26.84)	−8.466	<0.001
ESR (mm/h)	41.00 (33.00–48.00)	24.00 (17.00–32.00)	−6.184	<0.001
DD (mg/L)	2.66 (1.28–6.34)	0.66 (0.37–1.12)	−7.132	<0.001
PCT (ng/mL)	0.24 (0.10–1.34)	0.08 (0.05–0.13)	−5.727	<0.001
Ferritin (ng/mL)	311.90 (196.20–656.10)	120.10 (88.93–176.83)	−7.381	<0.001
Liver and kidney function parameters				
ALT(U/L)	49.00 (18.00–104.00)	14.50 (11.00–21.25)	−6.332	<0.001
AST(U/L)	47.00 (34.00–131.80)	29.00 (24.00–39.00)	−5.035	<0.001
LDH (U/L)	496.00 (315.00–672.00)	248.00 (215.00–297.00)	−6.717	<0.001
PA(mg/L)	101.00 (72.00–147.00)	114.50 (98.75–143.00）	−2.220	0.026
ALB(g/L)	34.80 (32.10–37.90)	39.80 (37.10–41.85)	−7.080	<0.001
TP(g/L)	61.20 (55.80–65.20)	66.20 (63.00–69.03)	−5.087	<0.001
Immune and cytokine parameters				
IgE (IU/mL)	192.00 (55.40–305.00)	142.00 (65.45–312.06)	−0.446	0.656
CD4	266.00 (172.00–387.00)	482.00 (307.00–669.50)	−5.732	<0.001
CD8	282.00 (185.00–356.00)	324.00 (235.50–454.00)	−2.815	0.005
CD19	213.00 (152.00–302.00)	248.00 (195.00–382.50)	−2.341	0.019
NK	76.00 (46.00–151.00)	108.00 (77.00–174.00)	−2.452	0.014
IL-4 (pg/mL)	2.23 (1.48–3.22)	1.49 (0.87–2.30)	−3.610	<0.001
IL-6 (pg/mL)	17.87 (7.56–31.45)	13.30 (6.94–21.55)	−1.582	0.114
IL-10 (pg/mL)	9.92 (5.39–15.67)	6.49 (4.37–10.38)	−3.300	0.001
γ-IFN (pg/mL)	13.09 (8.97–24.67)	5.62(2.75–10.65)	−5.598	<0.001

Data are presented as the median (25th–75th percentile).

ALT, alanine aminotransferase; AST, aspartate aminotransferase; LDH, lactate dehydrogenase; PAB, prealbumin; ALB, albumin; TP, total protein; CRP, C-reactive protein; ESR, erythrocyte sedimentation rate; PCT, procalcitonin; IL, interleukin; IFN-γ, interferon-gamma.

### 3.3 Radiologic characteristics

Comparisons of radiologic characteristics between the two groups were presented in Table 3. Chest CT scan was performed in all patients during the hospitalization, and 51 cased of NP were identified. The NP group had a significantly higher proportion of patients with bronchial cast formation, inflammatory bronchial stenosis, pleural effusion, and atelectasis compared to the NNP group (*P* *<* 0.001, *P* *<* 0.001, *P* *<* 0.001, *P* *<* 0.01, respectively). However, regarding to the distribution of involved lung lobes or the proportion of pulmonary consolidation, there were no statistically significant differences between these two groups.

**Table 3:** Chest imaging features of children with RMPP in the NP group and NNP group.

Radiological features	NP group (*n* = 51)	NNP group (*n* = 223)	*χ*^2^/*Z* value	*P* value
Pulmonary consolidation^a^			24.053	<0.001
Present	51 (100.00)	147 (65.92)		
Absent	0 (0.00)	76 (34.08)		
Bronchoscopic plastic bronchitis^a^			11.588	0.001
Yes	13 (25.49)	19 (8.52)		
No	38 (74.51)	204 (91.48)		
Number of inflamed lobes^b^			−1.789	0.074
1	38 (74.51)	120 (53.81)		
2	3 (5.88)	67 (30.04)		
3	2 (3.92)	24 (10.76)		
>3	8 (15.69)	12 (5.38)		
Pleural effusion^a^			81.174	<0.001
Present	48 (94.12)	58 (26.01)		
Absent	3 (5.88)	165 (73.99)		
Atelectasis^a^			7.990	0.005
Present	11 (21.57)	18 (8.07)		
Absent	40 (78.43)	205(91.93)		

^a^ Represented the *χ*^2^ test.

^b^ Represented the Mann–Whitney *U* test.

### 3.4 Multivariate analysis

Using NP status (0 = NNP, 1 = NP) as the dependent variable, and 26 variables showing statistical significance in univariate analysis as independent variables, a multivariable logistic regression model was constructed in the training set (*n* = 197). The logistic regression showed ESR, CD4^+^ cell count, NK cell count, IL-4 level, duration of fever, and presence of pleural effusion were the risk factors for NP caused by MPP. The Hosmer-Lemeshow test indicated good model fit (*χ*² = 3.057, *df* = 8, *P* = 0.931), failing to reject the null hypothesis of adequate fit. The VIF for the independent variables ranged from 1.021 to 1.310, all well below 10, indicating no significant multicollinearity concerns.

The partial regression coefficients and odds ratios (OR) for each independent variable were presented in Table 4. ESR, NK cell count, IL-4 level, duration of fever, and presence of pleural effusion demonstrated positive associations with NP risk. Specifically, each 1-unit increase in ESR (OR = 1.071) conferred an average 7.1% increase in NP risk; each 1-unit increase in NK cell count (OR = 1.011) conferred an average 1.1% increased risk; and each 1-unit increase in IL-4 (OR = 1.609) conferred an average 60.9% increased risk. For each additional day of fever duration (OR = 1.410), NP risk increased by an average of 41.0%. Children presenting with pleural effusion had 68.773 times the risk of developing NP compared to those without this finding. Conversely, CD4^+^ cell count exhibited a negative association with NP risk (OR = 0.995), with each 1-unit increase conferring an average 0.5% reduction in risk.

**Table 4:** Multivariable logistic regression analysis of independent predictors for NP in pediatric RMPP.

Variable	β	SE	Wald χ^2^ value	*P* value	OR value	95%CI
Intercept	−10.139	2.247	20.367	<0.001		
ESR	0.069	0.021	10.570	0.001	1.071	1.028–1.116
CD4	−0.005	0.002	8.483	0.004	0.995	0.991–0.998
NK	0.011	0.004	7.654	0.006	1.011	1.003–1.020
IL-4	0.475	0.213	4.961	0.026	1.609	1.059–2.444
Length of fever	0.344	0.120	8.265	0.004	1.410	1.116–1.782
Pleural effusion	4.231	1.017	17.322	<0.001	68.773	9.379–504.308

### 3.5 ROC curves and prediction nomogram

**Figure 2:**
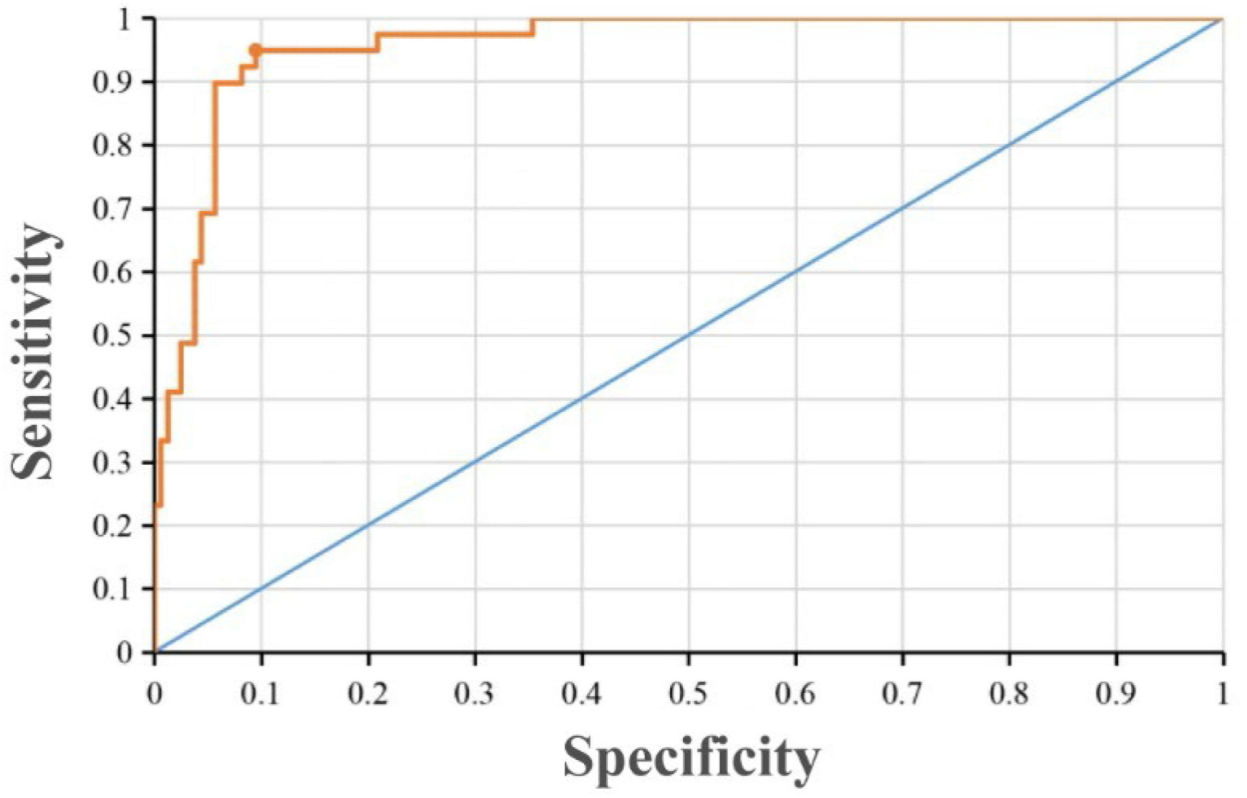
The ROC curve of the prediction model for NP. The area under the curve (AUC) was 0.957 (95% CI: 0.830–0.985) in the training set.

Based on the multivariable logistic regression equation, the prediction model for NP was established as follows: logit(P) = −10.139 + 0.069 X _ESR_−0.005 X _CD4+_ + 0.011 X _NK_ + 0.475 X _IL-4_ + 0.344 X _duration of fever_ + 4.231 X _pleural effusion_. The predictive validity of the NP probability was assessed using ROC curve analysis (Figure 2). The area under the curve (AUC) was 0.957 (95% CI: 0.830–0.985). The optimal probability cutoff point for NP diagnosis, determined by maximizing the Youden index (Youden index = sensitivity + specificity - 1), was 0.265. At this cutoff, the sensitivity was 0.949 and the specificity was 0.905. Thus, within the training set, a calculated probability greater than 26.5% indicated a diagnosis of NP, yielding optimal predictive performance.

**Figure 3:**
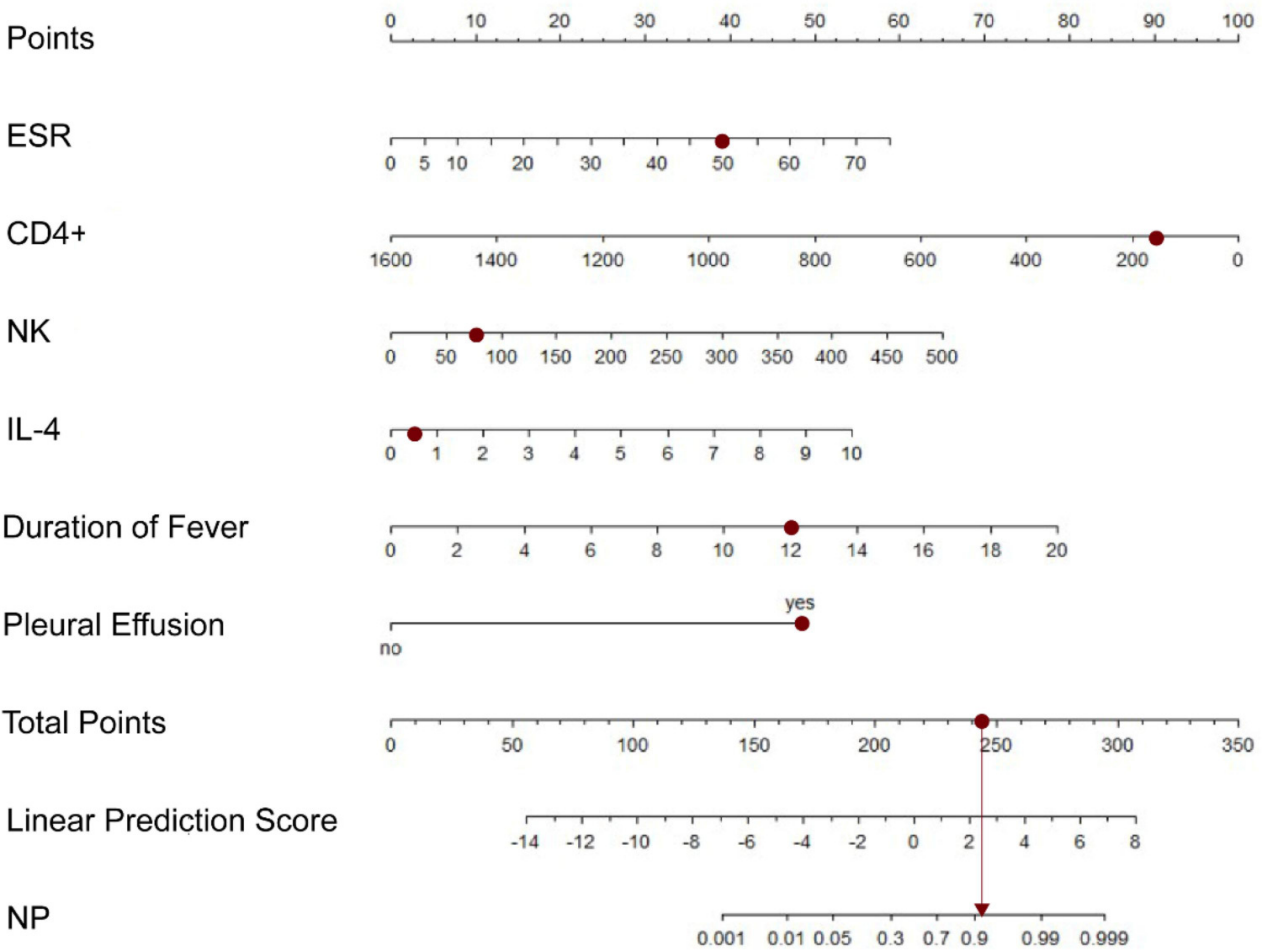
Nomogram for predicting the risk of NP in children with RMPP. The nomogram incorporates six independent predictors: ESR, CD4^+^ *T*-cell count, NK cell count, IL-4 level, duration of fever, and presence of pleural effusion.

Additionally, based on the results of the multivariable analysis, a nomogram was constructed to estimate the individual risk of progressing to NP in children with RMPP (Figure 3). These variables included ESR, CD4^+^ *T*-cell count, NK cell count, IL-4 level, duration of fever, and the presence of pleural effusion. The total points, derived from the sum of the points assigned to each predictor in the nomogram, correlated with the risk of developing NP. For illustration, a sample calculation is provided: a patient with RMPP exhibiting an ESR of 50 mm/h (assigned 39 points), a CD4^+^ count of 266 cells/μL (90 points), an NK cell count of 80 cells/μL (11 points), an IL-4 level of 0.59 pg/mL (5 points), a fever duration of 12 days (39 points), and pleural effusion (50 points), would accumulate a total score corresponding to an estimated NP probability of approximately 90.9%.

The model's generalizability was validated using the test set (*n* = 77). Applying the established regression equation, cases were predicted as NP-positive if the probability exceeded the 0.265 threshold. Comparison between predicted and actual NP status (Table 5) showed 87.01% concordance (67/77), with sensitivity 0.833, specificity 0.877, and Cohen's *κ* = 0.590. Although predictive accuracy decreased compared to the training set, the model retained satisfactory performance, demonstrating potential for clinical application.

**Table 5:** Predictive performance of the nomogram in the training and internal validation sets.

Group	Predictive performance		Total
	Positive	Negative	
NP	10	2	12
NNP	8	57	63
Total	18	59	77

## 4 Discussion

The post-COVID-19 era has witnessed a global epidemic surge of MP infections since 2023 (18). Notably, reports of RMPP have increased markedly worldwide, with the most pronounced rise observed across Asia (19). Contemporary research prioritizes identifying risk factors for severe pulmonary complications in RMPP, including bronchiolitis obliterans, plastic bronchitis, bronchopleural fistula, persistent atelectasis, and NP. This epidemiological shift underscores an urgent need for early identification of severe complications, notably NP, which manifests through a characteristic pathological sequence: initial pulmonary consolidation progresses to low-density lesions within 7 days, culminating in cavitary necrosis (20). Affected patients frequently develop effusion-associated pneumothorax and exertional dyspnea. The symptoms precipitated by minimal activity or exercise (21). Without timely intervention, NP may advance to life-threatening cardiorespiratory compromise (22).

Therefore, this retrospective observational study enrolled 274 pediatric patients with MPP during the epidemic surge period (July 2023 - June 2024). The different clinical characteristics between the NP patients and NNP patients were compared. According to the diagnostic criteria of NP, 51 cases were diagnosed as NP, while 223 were NNP. Similar age and gender distribution were observed in NP group and NNP group caused by MPP, which meant that age and gender would not be associated with the incidence of NP.

Moreover, we further identified that the duration of fever was significantly longer in children with NP than in those with NNP, a finding consistent with multiple domestic and international studies, further confirming that prolonged fever serves as a significant clinical predictor for the development of NP induced by RMPP (16, 19, 23). The pathogenesis of NP involves not only direct damage to lung tissue caused by pathogens and their toxins but is also closely associated with excessive activation of the host's immune-inflammatory response, leading to a massive release of inflammatory factors and subsequent secondary injury (23). During febrile episodes, increased body temperature elevates the basal metabolic rate, which can induce clinical manifestations such as tachycardia, tachypnea, and dehydration. These physiological alterations further disrupt immune homeostasis, while an exaggerated inflammatory response significantly exacerbates the progression of pulmonary necrosis (24). Meanwhile, pleural effusion emerged as another critical clinical predictor for NP, indicating that pulmonary inflammation has become severe enough to breach the parenchymal barrier and extend to the pleural space. The formation of effusion results from a robust inflammatory response. Its presence strongly implies equally severe or even more profound damage in deep lung tissues, serving as a potent risk indicator for NP (25). Multiple studies have consistently demonstrated a significantly higher incidence of pleural effusion in children with NP compared to those in the non-NP group.

As to laboratory data, a significant elevation in the ESR is strongly associated with a systemic hyperinflammatory response. The increase in ESR is primarily driven by elevated plasma levels of fibrinogen and immunoglobulins, and its marked elevation reflects a state of intense acute-phase reaction and highly activated immune status (25, 26). This sustained and intense inflammatory response further exacerbates pulmonary tissue destruction, promotes microthrombus formation, and may even lead to ischemic necrosis (25, 27). Thus, ESR may serve as a valuable predictive indicator for the development of NP. Infection with MP triggers a potent immune response in the host, leading to alterations in cellular antigenicity and resulting in immune dysregulation (28). Research on this aberrant immune status has primarily focused on humoral and cellular immunity (28). T lymphocytes play a central regulatory role in the cellular immune response, with CD4^+^ T cells, as helper T cells, being crucial in defending against intracellular pathogens (29). They promote phagocyte-mediated defense, assist in T- and B-cell-mediated immune responses, and help suppress excessive immune activation, thereby maintaining immune balance (30). A significant decrease in CD4^+^ *T* cells indicated functional impairment and reflects disordered cellular immunity. At the level of immune mediators, IL-4 is primarily secreted by Th2 cells and modulates the humoral immune response following infection (31). Overproduction of IL-4 reflects a shift toward Th2-polarized immunity, which can suppress the Th1-dominated response required for effective clearance of intracellular pathogens (32, 33). More importantly, a dominant Th2 response can promote eosinophil infiltration into lung tissue, activate B cells and antibody production, and may exacerbate tissue fibrosis (34, 35). Simultaneously, NK cells also play a key immunoregulatory role, not only through direct cytotoxicity against target cells but also via fine-tuning of immune responses (36). Impaired function or reduced numbers of NK cells may hinder viral clearance and disrupt immune regulation; conversely, overactivation of NK cells can contribute directly to lung tissue damage through excessive release of inflammatory factors and cytotoxic effects (37–39). Thus, the functional dysregulation of CD4^+^ T cells, elevated IL-4 levels, and abnormal NK cell activation collectively constitute the deep immunopathological basis for predicting the risk of NP.

During the management of RMPP, the presence of persistent high fever, significantly elevated ESR, abnormal CD4^+^ T-cell and NK cell counts, and increased IL-4 levels should raise clinical suspicion for NP. In such cases, dynamic pulmonary imaging surveillance is recommended to facilitate early detection of necrotic changes. This study developed a simple and practical nomogram that may serve as an effective tool for early identification of NP, thereby supporting timely intervention and rational treatment administration.

Furthermore, this study has several limitations that should be acknowledged. First, the retrospective design and inherent data constraints present certain challenges. The assessment of nutritional status was limited by incomprehensive recording of height and weight, preventing precise BMI stratification across the cohort, though clinical records confirmed no documented cases of malnutrition. Additionally, while all included patients had negative preliminary HIV screenings, the absence of routine confirmatory testing remains a consideration despite the low local HIV prevalence. Furthermore, the prediction model developed in this study lacks validation in an external cohort, and its generalizability is constrained by the single-center retrospective nature of the research. Therefore, future prospective, multi-center studies with larger sample sizes are warranted to validate these findings and improve the early prediction of necrotizing pneumonia.

## 5 Conclusion

This study developed an early prediction nomogram for NP in children with RMPP based on four clinical variables, namely persistent high fever, elevated ESR, abnormal CD4^+^ *T*-cell and NK cell counts, and increased IL-4 levels. As a simple and practical tool, this model could assist clinicians in identifying high-risk children with RMPP who may progress to NP, thereby facilitating timely intervention and ultimately improving treatment outcomes.

## Data Availability

The raw data supporting the conclusions of this article will be made available by the authors, without undue reservation.
